# A Tensor-Based Framework for rs-fMRI Classification and Functional Connectivity Construction

**DOI:** 10.3389/fninf.2020.581897

**Published:** 2020-11-30

**Authors:** Ali Noroozi, Mansoor Rezghi

**Affiliations:** Department of Computer Science, Tarbiat Modares University, Tehran, Iran

**Keywords:** Alzheimer's disease (AD) classification, functional connectivity, tensor, high order singular value decomposition, dimension reduction

## Abstract

Recently, machine learning methods have gained lots of attention from researchers seeking to analyze brain images such as Resting-State Functional Magnetic Resonance Imaging (rs-fMRI) to obtain a deeper understanding of the brain and such related diseases, for example, Alzheimer's disease. Finding the common patterns caused by a brain disorder through analysis of the functional connectivity (FC) network along with discriminating brain diseases from normal controls have long been the two principal goals in studying rs-fMRI data. The majority of FC extraction methods calculate the FC matrix for each subject and then use simple techniques to combine them and obtain a general FC matrix. In addition, the state-of-the-art classification techniques for finding subjects with brain disorders also rely on calculating an FC for each subject, vectorizing, and feeding them to the classifier. Considering these problems and based on multi-dimensional nature of the data, we have come up with a novel tensor framework in which a general FC matrix is obtained without the need to construct an FC matrix for each sample. This framework also allows us to reduce the dimensionality and create a novel discriminant function that rather than using FCs works directly with each sample, avoids vectorization in any step, and uses the test data in the training process without forcing any prior knowledge of its label into the classifier. Extensive experiments using the ADNI dataset demonstrate that our proposed framework effectively boosts the fMRI classification performance and reveals novel connectivity patterns in Alzheimer's disease at its early stages.

## 1. Introduction

Alzheimer's disease (AD) is a progressive neurodegenerative disorder with a long pre-morbid asymptomatic period, which affects millions of elderly individuals worldwide (Caselli et al., 2004). It is predicted that the number of affected people will double in the next 20 years, and 1 in 85 people will be affected by 2050 (Brookmeyer et al., 2007). The predominant clinical symptoms of AD include a decline in some important brain cognitive and intellectual abilities such as memory, thinking, and reasoning. Early detection is important for possible delay of the progression of mild MCI to moderate and severe stages (Folch et al., 2016). However, diagnosis of MCI is difficult due to its mild symptoms of cognitive impairment, causing most computer-aided diagnosis to achieve lower-than-desired performance (Musha et al., 2013; Li R. et al., 2018). Precise diagnosis of AD, especially in its early warning stage, that is, early Mild Cognitive Impairment (eMCI), enables treatments to delay or even avoid such disorders.

In recent years, medical imaging techniques such as positron emission tomography (PET) (Chandra et al., 2019), electroencephalography (EEG) (Bi and Wang, 2019), computed tomography (CT) scan (Ozdemir et al., 2019; van de Leemput et al., 2019), intracoronary imaging (Gao et al., 2019), and functional magnetic resonance imaging, which is a non-invasive brain imaging technique (fMRI) (Golby et al., 2005), have been used in order to analyze and detect disorders within body and brain (Zhang et al., 2012; Han et al., 2013). Due to high spatial resolution, fMRI is vastly used among researchers in order to monitor brain activities, especially in AD and all its stages in which detecting abnormalities within small brain regions is essential (Dennis and Thompson, 2014). An fMRI sample is naturally a 4D tensor consisting of 3D time-varying voxels, and each voxel contains an intensity value that is proportional to the strength of the *B*lood *O*xygenation *L*evel *D*ependent (BOLD) signal, which is a measure of the changes in blood flow to estimate the activity of different brain regions. Resting-state fMRI (rs-fMRI) is an fMRI technique in which the patient is asked to rest during the whole scan and it focuses on the low-frequency (< 0.1 *Hz*) oscillations of BOLD signal presenting the underlying neuronal activation patterns of brain regions. rs-fMRI is usually used in order to analyze brain diseases like AD or Autism (Leonardi et al., 2013; Kazeminejad and Sotero, 2019; Nguyen et al., 2019). Different toolboxes such as GraphVar (Waller et al., 2018), Graph CNN (Zhang et al., 2019), and BrainNetClass (Zhou et al., 2020) are also developed to aid this cause.

**Figure 1:**
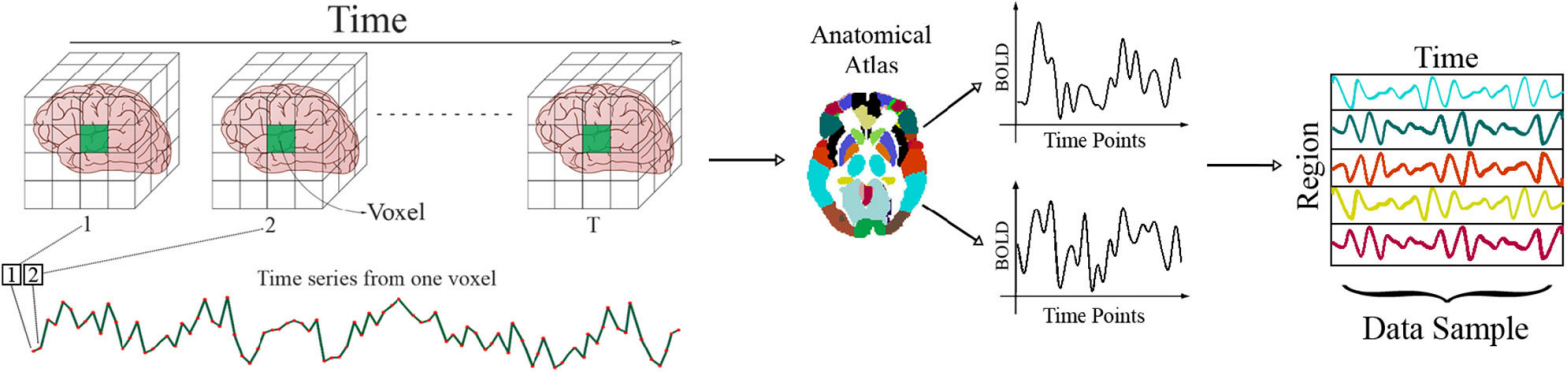
The process of obtaining $X_{i}\in {\mathbb{R}}^{T\times R},i=\left\{1,2,\cdots \;,S\right\}$.

Since each fMRI series consists of hundreds of thousands of voxels, which are often highly correlated with the surrounding voxels in the brain volume, parcellation of the brain for further analysis has moved toward the use of anatomical atlases. These atlases are strictly defined using anatomical features of the brain like locations of common gyri and do not rely on any functional information. To generate data using an atlas-based approach, the BOLD signal from all voxels is averaged within each brain region called region of interest (ROI) (Stanley et al., 2013). By putting together the average time series for all the ROIs, the *i*th series becomes $X_{i}\in {\mathbb{R}}^{T\times R},i=\left\{1,2,\cdots \;,S\right\}$, in which *R*, *T*, and *S* are the number of ROIs, time points, and samples, respectively. This process is illustrated in Figure 1

There are two major studies associated with rs-fMRI data: finding common brain disorders caused by diseases such as AD, autism, schizophrenia, and so on, and more recently detecting patients with brain disorders using classification techniques (de Vos et al., 2018; Du et al., 2018). Due to the high dimensionality of data along with the nature of diseases such as eMCI, which does not show any reliable clinical symptoms, researchers have moved toward advanced machine learning techniques in order to achieve more reliable analysis (Cuingnet et al., 2011).

A powerful tool that is commonly used in order to achieve aforementioned goals is the functional connectivity (FC) network. Let *X*_*i*_ be the *i*th sample, its corresponding FC, and $\bar{X}$ is a *region* × *region* matrix in which ${\bar{x}}_{ij}$ represents the FC between the *i*th and *j*th ROI. Functional connectivity is an observable phenomenon quantifiable with measures of statistical dependencies such as correlations, coherence, or transfer entropy (Friston, 2011). Recent studies have shown that some brain disorders such as AD could alter the way some brain regions interact with each other. For example, compared with healthy subjects, AD patients have been found with decreased FC between the hippocampus and other brain regions, and MCI patients have been observed with increased FC between the frontal lobe and other brain regions (Dennis and Thompson, 2014).

FCs are also used as features in classification. So, instead of using *X*_*i*_ as the *i*th sample, its corresponding FC, that is, ${\bar{X}}_{i}$, is used as a feature. Common techniques for calculating FC, that is, simple statistical measures such as coherence and Pearson correlation, allow for different ambiguities (Smith, 2012; Reid et al., 2019). And since brain alterations in early MCI are tiny, more sophisticated and computationally expensive methods such as partial correlation (Li Y. et al., 2018; Pervaiz et al., 2020), high-order networks (Chen et al., 2016), and spectral clustering (Liu et al., 2018) are required in order to achieve a better FC. The computational cost of a sophisticated FC is usually high and also its quality affects the performance of the learning process massively. Also, since the conventional classifiers like *S*upport *V*ector *M*achine (SVM) or k-NN works on data in vector format, these matrix features should be vectorized in order be fed to these classifiers. This vectorization leads to high-dimensional vectors that produce poor performance due to the phenomenon known as the curse of dimensionality. Alongside the curse of dimensionality, vectorization also destroys potential information embedded in the structure of data. This problem has been studied especially in image data in which vectorization destroys the spatial relations within an image (Ahmadi and Rezghi, 2020).

In this paper, based on high-order tensor decomposition, we have created a framework in which the aforementioned goals, that is finding a general FC and detecting a disorder through classification, could be achieved via a single *H*igh-*O*rder *S*ingular *V*alue *D*ecomposition (HOSVD) of each class. Here based on latent variables obtained by HOSVD, a general representative pattern of FC for eMCI and normal controls are obtained. As it was mentioned before, finding a proper FC is a challenging task. Obtaining an FC via the proposed method is not only fast and straightforward, but also very accurate. The majority of connectivity patterns detected by this method have been observed and studied in several separated types of research (cited in the experimental studies section), which show the reliability and power of the proposed method. Along with these connections, we have also detected novel connectivities especially regarding the cerebellum, which is usually discarded in the analysis of AD.

The proposed classifier is also much faster than the state-of-the art classifiers, and also uses the obtained rs-fMRI data directly in the classification process rather than calculating its corresponding FC matrix. Being able to feed *X*_*i*_s directly to the classifier saves us from a lot of problems related to calculating the proper FC. It also shows that the proposed classifier is strong enough to detect tiny alterations, which other state-of-the-art methods rely on finding FC to highlight.

To verify our approach, we conducted an extensive experimental study on rs-fMRI data from the benchmark dataset ADNI. As will be seen, the results demonstrate the effectiveness and advantages of our method. Specifically, the proposed framework not only grants us superior classification accuracy to that from other methods, but it is also much faster and more stable against different data selection schemes. We have also confirmed our achieved general FC matrix using empirical data on the eMCI and normal FC patterns.

## 2. Related Works

As it was mentioned previously, obtaining and classifying FC matrices have become the dominant approach toward eMCI analysis. Variety of methods such as pairwise Pearson's correlation coefficient, sparse representation (Jie et al., 2013), and *S*parse *I*nverse *C*ovariance *E*stimation (SICE) (Huang et al., 2010) exist to obtain an FC. While the first two are easy to understand and can capture pairwise functional relationship based on a pair of ROIs, the latter can account for more complex interactions among multiple ROIs, but the estimation of partial correlation involves an inversion of a covariance matrix, which may be ill posed due to the singularity of the covariance matrix. These methods result in vastly different networks (Du et al., 2018). On the other hand, computing the correlations, based on the entire time series of fMRI data simply measures the FC between ROIs with a scalar value, which is fixed across time. This actually implicitly hypothesizes the Stationary interaction patterns among ROIs, which will result in a static functional connectivity (sFC). As a result, this method may overlook the complex and dynamic interaction patterns among ROIs, which are essentially time-varying (since the phase is not locked for every subject). In order to overcome this issue, Non-stationary methods have been proposed, which result in more complex networks and also known as dynamic functional connectivity (dFC) (Leonardi and Van De Ville, 2015; Kam et al., 2019). The most common and straightforward way to investigate dFC is using windowed FC, which consists of calculating a given FC measure, for example, the Pearson correlation coefficient, over consecutive windowed segments of the data (Zalesky et al., 2014). Although such an analysis seems straightforward, there are also pitfalls associated with it, which may cause in a non-accurate FC network (Hindriks et al., 2016).

In the following, we briefly discuss two state-of-the-art eMCI classification techniques belonging to these two paradigms:

**Kernel compact SICE** (**K**−**SIEC**): SICE matrix have proven itself to be one of the best sFC models (Huang et al., 2010; Ng et al., 2013; Colclough et al., 2018; Foti and Fox, 2019), which is extracted via the following optimization:

(1) $$\begin{array}{l} S^{*}=\text{arg}\underset{S≻0}{\text{ max }}\text{ log}\left(\text{det}\left(S\right)\right)-\text{tr}\left(CS\right)-\lambda {∥S∥}_{1} \end{array}$$

where *C* is the sample-based covariance matrix; det(Δ), tr(Δ), and ∥.∥_1_ denote the determinant, trace, and the sum of the absolute values of the entries of a matrix, respectively. In classification with FC features, the vectorized SICE of each sample is used (Leonardi et al., 2013). The occurrence of the curse of dimensionality and losing useful information contained in the SICE matrices [like symmetric positive definite (SPD) property] are two main drawbacks of this vectorization approach. As an inverse covariance matrix, an SICE matrix is SPD. This inherent property restricts SICE matrices to a lower dimensional Riemannian manifold rather than the full dimensional Euclidean space. This property allows some SPD manifold-based distances, like log-Euclidean distance (Arsigny et al., 2006) and Root Stein divergence (Sra, 2012) to be employed in kernel-based PCA to extract a compact representation of brain network (Zhang et al., 2015). The power of this method resides in a massive dimension reduction of SICE using its SPD property. The performance of this method also heavily relies on the choice of sparsity parameter λ for SICE calculations and the number of top eigenvectors *m*.

**High−order networks** (**HON**): This method which is proposed in Chen et al. (2016) belongs to non-stationary paradigm and uses the so-called high-order networks as features for classification purposes. It uses the sliding-window technique in order to split the time series into smaller pieces and then find the relation between them (Chang and Glover, 2010; Handwerker et al., 2012; Allen et al., 2014). Let $x_{i}^{\left(l\right)}\left(k\right)\in {\mathbb{R}}^{N}$ denotes the *k*th segment of the *i*th region in the *l*th sample. For each sample, a network with nodes $x_{i}^{\left(l\right)}\left(k\right)$ could be constructed, in which its edge weights are obtained as

$$C_{ij}^{\left(l\right)}\left(k\right)=\text{corr}\left(x_{i}^{\left(l\right)}\left(k\right),x_{j}^{\left(l\right)}\left(k\right).\right)$$

Here, the weight $C_{ij}^{\left(l\right)}\left(k\right)$ represents the pairwise Pearson's correlation coefficients between the *i*th and the *j*th ROIs of the *l*th subject using the *k*th segment of subseries. Now

$$y_{ij}^{\left(l\right)}=\left[C_{ij}^{\left(l\right)}\left(1\right),C_{ij}^{\left(l\right)}\left(2\right),\cdots \;,C_{ij}^{\left(K\right)}\left(1\right)\right]\in {\mathbb{R}}^{K}$$

represents the similarity of the *i*th and *j*th ROIs of the *l*th sample in all segments. For each *l* by considering $y_{ij}^{\left(l\right)}$ as nodes of a networks with weights

$$H_{ij,pq}^{\left(l\right)}=\text{corr}\left(y_{ij}^{\left(l\right)},y_{pq}^{\left(l\right)}\right)$$

a higher-order network is obtained for each sample. Here for each pair of correlation time series *y*_*ij*_ and *y*_*pq*_, $H_{ij,pq}^{\left(l\right)}$ indicates how the correlation between the *i*th and the *j*th ROIs influences the correlation between the *p*th and the *q*th ROIs. So for each sample its higher-order networks $\left\{H_{ij,pq}^{\left(l\right)}\right\}$ will be a matrix with size *R*^4^ × *R*^4^(*R* is the number of regions), which will lead to a large-scale high-order FC network, containing at least thousands of vertices and millions of edges. In order to overcome this issue, the correlation time series within each subject are grouped into different clusters. Then, the correlation computations are carried out between the means of clusters. After reducing the network size, the weighted-graph local clustering coefficients is used to select the key features for each network and then an SVM classifier is trained in order to classify the obtained features. As a result of constructing a high-order network, the notion of a physical ROI become vague and thus such networks are not preferable choices in order to analyze functional connectivities.

Our method overcomes the dynamic-stationary problem of FC construction by working in HOSVD-based domain, which considers the dynamic nature of data and is much more sophisticated than using a windowed FC. The obtained FC also considers all subjects within a class simultaneously, rather than calculating FC for each subject separately that highlights common patterns in a class and eliminates possible outliers within data. The proposed framework also does not require any FC calculations for classification, which is a major advantage since finding a proper FC for each subject might be a very challenging task.

Multilinear approaches have been used before in order to analyze fMRI data. For example, Park (2011) uses multilinear PCA to classify fMRI data by Subject and Motor Task. Ozdemir et al. (2017) and Al-sharoa et al. (2018) use tensor decomposition and clustering techniques for analyzing brain connectivity networks and proves the dynamic nature of rs-fMRI. Recently, Ma et al. (2016) and He et al. (2017) proposed a multilinear method for voxel-wise analysis of rs-fMRI, which is used in order to detect late AD and some other diseases. Leonardi and Van De Ville (2013) considers dynamic whole-brain FC estimated from fMRI data acquired during alternating epochs of resting and watching of movie excerpts, and uses HOSVD in order to retrieve connectivity maps with associated time courses and subject loadings. This method uses the sliding-window technique in order to estimate the dynamic connectivity matrix for each subject, and then it constructs a 3-way tensor $R\in {\text{R}}^{C\times T\times T}$, by stacking the dynamic correlation matrices *R* of all subjects. Considering the HOSVD of *R*, this method obtains a matrix columns of which could be interpreted as group connectivity maps. There are similarities between this method and ours since they both take advantage of HOSVD. But our framework introduces major advantages such as (1) our framework does not require any FC calculations for its classifier. And (2), it is able to work with rs-fMRI, which is harder due to less constraint status of subjects.

## 3. Notation and Preliminaries

Tensors can be considered as a generalization of vectors and matrices of high dimensions. We use calligraphic letters to denote the tensors, for example, (A,B). Let $A\in {\mathbb{R}}^{I_{1}\times I_{2}\times I_{3}}$ denote an order-3 tensor. Different “dimensions” of tensors are referred to as *modes*. We will use both standard subscripts and “MATLAB-like” notation to show tensor elements as follows:

$$A\left(i,j,k\right)=a_{ijk}.$$

A *fiber* is a subtensor, where all indices but one are fixed. For example, mode-2 fibers of A have the following form:

$$A\left(i,:,j\right)\in {\mathbb{R}}^{I_{2}}.$$

The mode-*n* product of an order-*M* tensor $A\in {\mathbb{R}}^{{𝕀}_{⊮}\times \cdots \times {𝕀}_{𝕄}}$ by a matrix $X\in {\mathbb{R}}^{K\times I_{n}}$ is defined as:

(2) $$\begin{array}{l} {\mathbb{R}}^{I_{1}\times \cdots \times I_{n-1}\times K\times I_{n+1}\times \cdots \times I_{M}}∋B={\left(X\right)}_{n}\cdot A, \end{array}$$

where,

$$b_{i_{1},\cdots \;,i_{M}}=\overset{I_{n}}{\underset{l=1}{\sum }}x_{i_{n,l}},a_{i_{1},\ldots ,i_{n-1},l,i_{n+1},\ldots ,i_{M}}.$$

This means that all mode-*n* fibers of A are multiplied by the matrix *X*. The notation (2) was suggested by De Silva and Lim (2008). An alternative notation was earlier given in De Lathauwer et al. (2000). ${\left(X\right)}_{n}\cdot A$ is the same as $A\times_{n}X$ in that system. The Frobenius norm of the order-*M* tensor A can be defined as $∥A∥=\sum_{i_{1},\cdots \;,i_{M}}a_{i_{1},\cdots \;,i_{M}}^{2}$.

### 3.1. Higher-Order Singular Value Decomposition

HOSVD is one common extension of singular value decomposition to the tensors (De Lathauwer et al., 2000). Using HOSVD, every order-M tensor $A\in {\mathbb{R}}^{I_{1}\times \cdots \times I_{M}}$ can be decomposed as:

(3) $$\begin{array}{l} A=\left(U^{\left(1\right)},\cdots \;,U^{\left(M\right)}\right)\cdot S \end{array}$$

where orthogonal matrices *U*^(*i*)^ are singular matrices of tensor A. Here, *U*^(*i*)^ is the left singular matrix of *A*^(*i*)^, in which its column vectors are the mode-*n* fibers of A. The core tensor S is a real tensor of the same dimensions as A and

$$S=\left(U^{{\left(1\right)}^{𝖳}},\cdots \;,U^{{\left(M\right)}^{𝖳}}\right)\cdot A$$

Although this core tensor is not diagonal as in the case of SVD of matrices, it satisfies the following conditions:

- **All orthogonality property**: Any two different slices along the same mode are orthogonal. This property of core tensor S is named as all orthogonality.
- **The ordering property**: The values $s_{j}^{k}=\left\|S\left(:,\cdots \;,:,j,:,\cdots \;,:\right)\right\|$, where *j* is in the *k*th mode of S, are named mode-*k* singular values of A. It can be shown that for every *k*
  (4) $$\begin{array}{l} s_{1}^{k}\geq s_{2}^{k}\geq \cdots \geq s_{n}^{k}\geq 0,\;k=0,\cdots \;,M, \end{array}$$
  are equal to the singular values of the matrix *A*^(*k*)^. This means that the norms of the slices along every mode are ordered.
- **Oscillation**: It can be shown that as the indices increase, the singular vectors of each mode shows more oscillation. Based on this property, it can be shown that noises and outliers within the data are transferred into these high oscillation parts (Rezghi, 2017). Based on this fact and also the ordering property, the truncated version of HOSVD can be deployed as a noise reduction and compression tool (Lv and Wang, 2019).

The ordering property (4) demonstrates that, in the same way as matrices, singular values measure the *energy* of the tensor. So, it is easy to see that the energy of core tensor S focused on the elements of S with small indices. This property of HOSVD (similar to SVD) is very useful in the applications that encounter denoising problems. So, if $U_{k_{l}}^{l}$ contains the first *k*_*l*_ singular matrix and $\hat{S}=S\left(1:k_{1},\cdots \;,1:k_{M}\right)$, the following truncated HOSVD:

$$\hat{A}={\left(U_{k_{1}}^{\left(1\right)},\cdots \;,U_{k_{M}}^{\left(M\right)}\right)}_{1:M}\cdot \hat{S},$$

is a rank-(*k*_1_, ⋯ .*k*_*M*_) approximation of A. Although this is not an optimal rank-(*k*_1_, ⋯ .*k*_*M*_) approximation of A, it is still a good approximation and we have:

$$∥A-\hat{A}∥=\overset{M}{\underset{i=1}{\sum }}\overset{r_{i}}{\underset{j=k_{i}+1}{\sum }}s_{j}^{{\left(i\right)}^{2}},$$

where *r*_*i*_ is the rank of *A*^*i*^ (De Lathauwer et al., 2000).

## 4. Proposed fMRI Analysis Framework Based on HOSVD

In this section, which is divided into three subsections we first tackle the problem of classification, that is, designing a discriminant function that could predict the label of an unknown test subject. The second part describes a technique, which would enhance the designed classifier and the third part is allocated to find a general connectivity network for each class (e.g., eMCI subjects). All three aforementioned goals, that is, classification and its enhancement and finding a general FC for each class, evolve around a single HOSVD of each class, which provides us with basis for each mode (*time*, *region*, and *sample*) and enables us to capture the essence of each feature in a few low dimensional slices. We will use the obtained low-dimensional bases along the sample and region mode in order to design our discriminant function and obtain the general FC. The enhancement technique also comes from HOSVD characteristics, which enables us to involve test samples in the training process without forcing any a priori knowledge into the classifier.

### 4.1. eMCI Classification

Let tensors $X^{\left(i\right)}\in {\mathbb{R}}^{T\times R\times S_{i}}$ consists of normal and eMCI data for *i* = 1,2, respectively. Here *S*_1_, *S*_2_ are the number of normal and eMCI samples. For tensor $X^{\left(i\right)}$, the decomposition

(5) $$\begin{array}{l} X^{\left(i\right)}=\left(U^{\left(i\right)},V^{\left(i\right)},W^{\left(i\right)}\right)\cdot S^{\left(i\right)}, \end{array}$$

is known as HOSVD, where orthogonal matrices *U*^(*i*)^ ∈ ℝ^*T*×*T*^, *V*^(*i*)^ ∈ ℝ^*R*×*R*^, and $W^{\left(i\right)}\in {\mathbb{R}}^{S_{i}\times S_{i}}$ are known as modes-1,2,3 singular matrices of $X^{\left(i\right)}$, and $S^{\left(i\right)}$ is the corresponding core tensor (Rezghi, 2017). Here, *U*^(*i*)^ is a base of all mode-1 fibers $X^{\left(i\right)}\left(:,l,k\right)$, which indicates the behavior of *l*th region of the *k*th sample of the *i*th class in all time points. Also *V*^(*i*)^ is a base of all mode-2 fibers X(l,:,k), which indicates the behavior of all regions of *l*th sample of the *i*th class in the *k*th time. Due to the properties of HOSVD inherited from svd, the first columns of the *k*th singular matrix (*k* = 1, 2, 3) have more ability in construction of main parts of *k*th fibers (Rezghi, 2017). Therefore, a suitable dimension reduction would be to project the mode-1 and mode-2 fibers into space spanned by the first $k_{1}^{i}$ and $k_{2}^{i}$ singular vectors of modes-1,2, which will be denoted by $U_{k_{1}^{i}}^{\left(i\right)}$ and $V_{k_{2}^{i}}^{\left(i\right)}$, respectively. This dimension reduction could be done as:

(6) $$\begin{array}{l} {\mathbb{R}}^{k_{1}\times k_{2}\times S_{i}}∋{\bar{X}}^{\left(i\right)}={\left(U_{k_{1}^{i}}^{\left(i\right)𝖳},V_{k_{2}^{i}}^{𝖳}\right)}_{1,2}\cdot X^{\left(i\right)} \end{array}$$

It is clear that this reduction could be done separately on each mode without the need to fold any of them. This means that the structural integrity of data is preserved during the dimension reduction process, which is a key aspect in our work. It has been shown that even choosing relatively small values for $k_{1}^{1}$ and $k_{2}^{i}$ would result in a very good reconstruction error (Ahmadi and Rezghi, 2020).

Inspired by the structure of this reduction, in the following we present a tensor-based discriminant function. By HOSVD decomposition of $X^{\left(i\right)}$, the projected data ${\bar{X}}^{\left(i\right)}$ in Equation (6) becomes

$$\begin{array}{l} {\bar{X}}^{\left(i\right)}=\left(\left[\begin{array}{cc} I_{k_{1}^{i}} & 0 \end{array}\right],\left[\begin{array}{cc} I_{k_{2}^{i}} & 0 \end{array}\right],W^{\left(i\right)}\right)\cdot S^{\left(i\right)}\\ \;={\left(W^{\left(i\right)}\right)}_{3}\cdot S^{\left(i\right)}\left(1:k_{1},1:k_{2},:\right) \end{array}$$

So, each sample of the *i*th class in the reduced space has the following form:

$$\begin{array}{l} {\bar{X}}^{\left(i\right)}\left(:,:,k\right)={\left(W^{\left(i\right)}\left(k,:\right)\right)}_{3}\cdot S^{\left(i\right)}\left(1:k_{1}^{i},1:k_{2}^{i},:\right)\\ \;=\overset{S_{i}}{\underset{k^{\prime }=1}{\sum }}W^{\left(i\right)}\left(k,k^{\prime }\right)\cdot S^{\left(i\right)}\left(1:k_{1}^{i},1:k_{2}^{i},k^{\prime }\right). \end{array}$$

This means that each sample in the *i*th class could be represented as linear combination of the slices of the tensor ${\bar{S}}^{\left(i\right)}=S^{\left(i\right)}\left(1:k_{1}^{i},1:k_{2}^{i},:\right)$. So if a test data like *X* ∈ ℝ^*T*×*R*^ belongs to the *i*th class, it is natural to expect that its projected version into principle region and times spaces, spanned by $U_{k_{1}^{i}},V_{k_{2}^{i}}$, that is,

$$Z^{\left(i\right)}={\left(U_{k_{1}^{i}}^{\left(i\right)𝖳},V_{k_{2}^{i}}^{\left(i\right)𝖳}\right)}_{1,2}\cdot X$$

could be approximated well as a linear combination of the slices of the tensor ${\bar{S}}^{\left(i\right)}$ as follows:

(7) $$\begin{array}{l} Z^{\left(i\right)}\approx \overset{S_{i}}{\underset{k=1}{\sum }}\lambda_{k}^{i}{\bar{S}}^{\left(i\right)}\left(:,:,k\right). \end{array}$$

Based on this viewpoint, each test data *X* could be assigned to a class that its projected version has the best approximation in the form (7). Due to the importance of core tensor elements with small indices in the reconstruction of the signal part of data in comparison with its last parts, the small number $k_{3}^{i}<S_{i}$ of slices ${\bar{S}}^{\left(i\right)}\left(:,:,k\right)$ could be used in (7). In this viewpoint, each test data *X* would be assigned to the *l*th class, if $r_{l}={\text{min}}_{i=1,2}r_{i},$ where

(8) $$\begin{array}{l} r_{i}=\underset{\lambda^{i}}{\text{min}}\|Z^{\left(i\right)}-\overset{k_{3}^{i}}{\underset{k=1}{\sum }}\lambda_{k}^{i}{\bar{S}}^{\left(i\right)}\left(:,:,k\right)\|,\;\lambda^{i}=\left(\begin{array}{c} \lambda_{1}^{i}\\ \vdots \\ \lambda_{s_{i}} \end{array}\right) \end{array}$$

*r*_*i*_ shows the reconstruction error of the projected version of *X* in the *i*th class.

### 4.2. Enhancing the Classifier

Consider that the test data *X* is added to dataset $X^{\left(i\right)}$ of the *i*th class. So the new dataset will be $\tilde{X}$$\in {\mathbb{R}}^{T\times R\times \left(S_{i}+1\right)}$:

$$\begin{array}{l} {\tilde{X}}^{\left(i\right)}\left(:,:,1:S_{i}\right)=X^{\left(i\right)},\\ {\tilde{X}}^{\left(i\right)}\left(:,:,S_{i}+1\right)=X. \end{array}$$

If *X* belongs to the *i*th class, then in the decomposition of ${\tilde{X}}^{\left(i\right)}$, *X* would be able to reinforce all slices of the core tensor and singular matrices. And thus enhances the reconstruction ability of (8) that would lead into a lower reconstruction error for the test subject *X*. On the other hand, if *X* does not belong to the *i*th class, HOSVD would naturally consider it as noise [based on ordering property (4)], since *X* is not similar to other samples and thus does not play a key role in reconstructing them, so its effect would be on the last slices of the core tensor and singular matrices, that is, slices with higher indices that are ignored in reconstruction (8).

**Figure 2:**
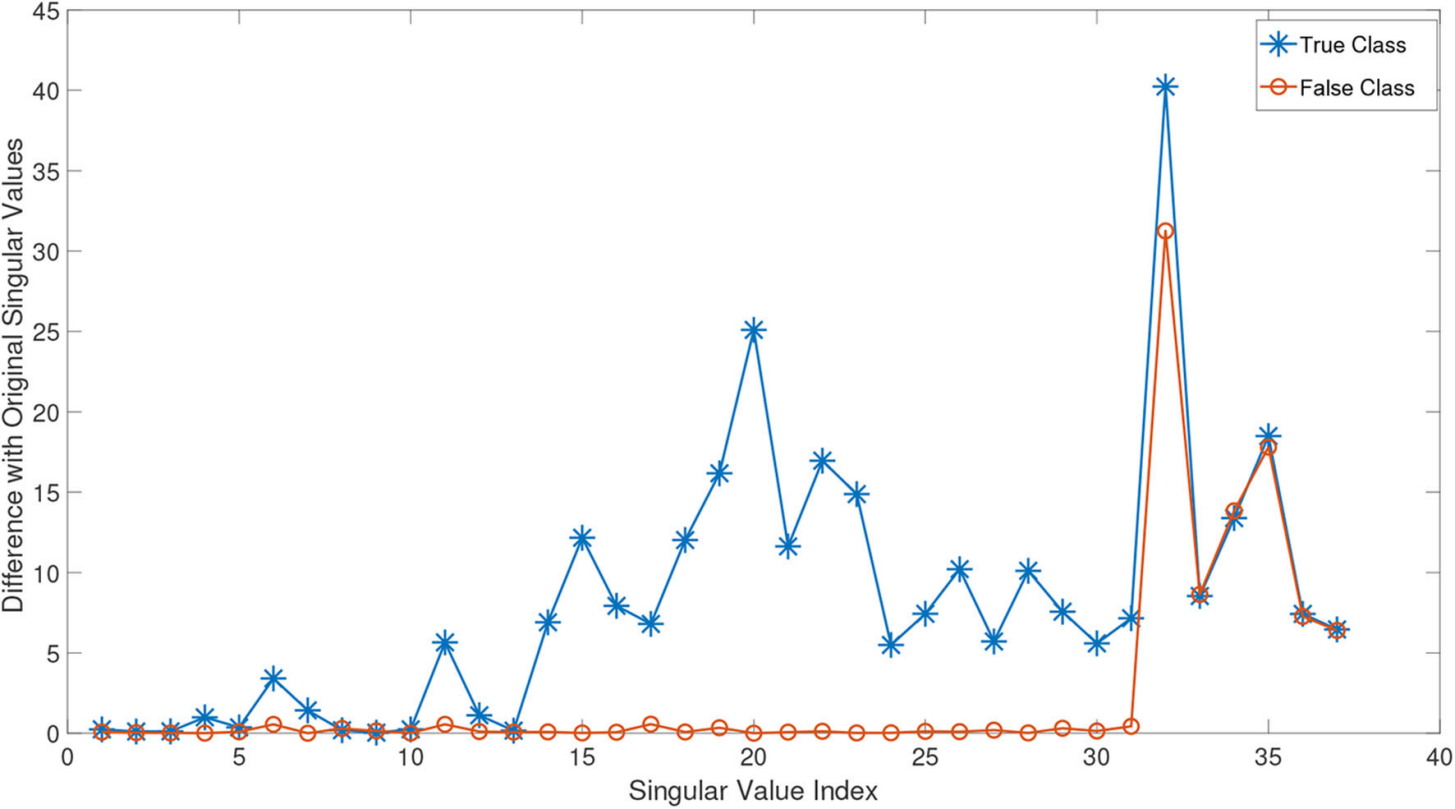
The absolute value difference of the third mode singular values of normal and eMCI data with and without involving test data in construction of HOSVD.

In order to better demonstrate this effect, we conducted the following experiment: we randomly chose a test subject *X*^*n*^ from the class of normal subjects in ADNI dataset (this dataset is explained in detail in the experimental study section). The remaining normal samples are then gathered in a tensor $X^{\left(1\right)}\in {\mathbb{R}}^{130\times 116\times 37}$. By adding *X*^*n*^ to this tensor, we obtained the incremented tensor ${\tilde{X}}^{\left(1\right)}\in {\mathbb{R}}^{130\times 116\times 38}$. We compute the HOSVD of these two tensors and plot the absolute mode-3 differences in Figure 2. As can be seen in this figure, since *X*^*n*^ belongs to the normal class, it effectively changes almost all singular values and so could improve the approximation in Equation (8). Then we randomly select an eMCI sample *X*^*e*^ and add it to $X^{\left(1\right)}$ to construct another incremented version of it. The orange line in equation (Figure 2) shows the absolute mode-3 differences between these two tensors. It can be observed that adding an eMCI subject to the class of normal subjects only affected the last singular values and have a very low impact on the first singular values.

It can be concluded that adding unknown labeled test data to all classes before the basis extraction process would heavily impact the true class bases, and it has a rather negligible or in ideal case zero impact on the bases of other classes. As a result, after extracting the basis for each class in this manner, the reconstruction error (Equation 8) would be lower for the true class. Note that in the training process, the test data are added to all classes and they are uninformed of its label. Thus, no a priori knowledge is sneaked into the decision-making process. Algorithm (1) summarizes the proposed classification method.

**Algorithm 1:** Tensor-based classification method

1) **Input**: Normal train data $X^{\left(1\right)}$, eMCI train data, $X^{\left(2\right)}$
$k_{j}^{i},i,j=1,2$.
Test data *X*
2) Construct ${\tilde{X}}^{\left(i\right)}$ for *i* = 1, 2, by adding *X* to both tensors.
3) Compute $U_{k_{1}^{i}},V_{k_{2}^{i}}$ and $S\left(1:k_{1}^{i},1:k_{2}^{i},:\right)$ of ${\tilde{X}}^{\left(i\right)}$, for i = 1,2.
4) Compute $Z^{\left(i\right)}={\left(U_{k_{1}^{i}}^{\left(i\right)T},V_{k_{2}^{i}}^{\left(i\right)T}\right)}_{1,2}\cdot X$, *i* = 1, 2.
5) Comput *r*_1_, *r*_2_ from (8)
6) Assign *X* to class *l*, if $l=\text{arg }\underset{i}{\text{min}}\left\{r_{i}\right\}$

### 4.3. General Functional Connectivity

In the *i*th class, which is represented by $X^{\left(i\right)}$, the slice $X^{\left(i\right)}\left(:,l,:\right)$ denotes the behavior of *l*^*th*^ region of all samples in all times. This slice could be considered as a feature for the *l*^*th*^ region of the *i*th class, so each region is represented as a Times-sample feature matrix. By the properties of singular matrices in modes-1,3, and for appropriate values $k_{1}^{i},k_{3}^{i}$, each region X(:,l,:) could be reduced in both time and sample features separately based on mode-1 and mode-3 truncated singular matrices $U_{k_{1}^{i}}^{\left(i\right)}$ and $W_{k_{3}}^{\left(i\right)}$ as follows:

(9) $$\begin{array}{l} Y^{\left(i\right)}\left(:,l,:\right)={\left({U_{k_{1}^{i}}^{\left(i\right)}}^{𝖳},{W_{k_{3}}^{\left(i\right)}}^{𝖳}\right)}_{1,3}\cdot X^{\left(i\right)}\left(:,l,:\right). \end{array}$$

Here $Y^{\left(i\right)}\left(:,l,:\right)$ denotes a reduced version of $X^{\left(i\right)}\left(:,l,:\right)$ into space spanned by $U_{k_{1}^{i}}^{\left(i\right)}$ and $W_{k_{3}}^{\left(i\right)}$ in modes-1,3. So,

(10) $$\begin{array}{l} {\mathbb{R}}^{k_{1}^{i}\times R\times k_{3}^{i}}∋Y^{\left(i\right)}={\left({U_{k_{1}^{i}}^{\left(i\right)}}^{𝖳},{W_{k_{3}^{i}}^{\left(i\right)}}^{𝖳}\right)}_{1,3}\cdot X^{\left(i\right)} \end{array}$$

denotes all reduced regions of the *i*th class. By this structure and substituting the HOSVD decomposition of $X^{\left(i\right)}$ in (10), we obtain

$$\begin{array}{l} Y^{\left(i\right)}=\left(\left[\begin{array}{cc} I_{k_{1}^{i}} & 0 \end{array}\right],V,\left[\begin{array}{cc} I_{k_{3}^{i}} & 0 \end{array}\right]\right)\cdot S^{\left(i\right)}\\ \;={\left(V\right)}_{2}\cdot S^{\left(i\right)}\left(1:k_{1}^{i},:,1:k_{3}^{i}\right) \end{array}$$

thus

(11) $$\begin{array}{l} Y^{\left(i\right)}\left(:,k,:\right)=\overset{R}{\underset{k^{\prime }}{\sum }}V^{\left(i\right)}\left(k,k^{\prime }\right){\bar{C}}^{\left(i\right)}\left(:,k^{\prime },:\right)\\ \begin{array}{l} \;={\left(V^{\left(i\right)}\left(k,:\right)\right)}_{2}\cdot C^{\left(i\right)} \end{array} \end{array}$$

in which

$${\mathbb{R}}^{k_{1}^{i}\times R\times k_{3}^{i}}∋C^{\left(i\right)}=S\left(1:k_{1}^{i},:,1:k_{3}^{i}\right).$$

Equation (11) shows that the reduced version of each region in the *i*th class could be written as the linear combinations of mode-2 slices of $C^{\left(i\right)}$. So the coefficients of slices in this linear combination could be considered as a new feature for the *l*^*th*^ region of the *i*th class. Also as we mentioned before, the first slices are better than the last ones to reflect the principle properties of the data. So for appropriate $k_{3}^{i}$ we could select only the first coefficients in (11) as new features for the *l*^*th*^ region. Mathematically, this means each region in the *i*th class could be represented by a new feature vector $V\left(l,1;k_{3}^{i}\right)\in {\mathbb{R}}^{k_{3}^{i}}$.

This approach has two main benefits: (1) each region could be represented only by a vector with size $k_{3}^{i}$ instead of a large time-sample matrix, and (2) the bases for each region is obtained in an HOSVD-based domain that is similar to Fourier frequency domain; but unlike Fourier, this transformation to HOSVD-domain is data dependent and hence the time-varying nature of rs-fMRI signals (2) would be taken into consideration (Rövid et al., 2013; Ozdemir et al., 2017). After representing each region with a single low dimensional vector, variety of methods such as SICE and other mentioned similarity measures could be deployed in order to construct a general FC for each class.

## 5. Experimental Study

### 5.1. Data Acquisition and Experimental Settings

RS-fMRI data of early MCI and NC patients were downloaded from ADNI website^1^. After removing subjects that had problems in the preprocessing steps, 44 eMCI and 38 NC subjects remained. The IDs of the 82 (38 NC and 44 early MCI) subjects are provided in the Supplementary Material.

The data are acquired on a 3-T (Philips) scanner with TR/TE set as 3,000/30 ms and flip angle of 80. Each series has 140 volumes, and each volume consists of 48 slices of image matrices with dimensions 64 × 64 with voxel size of 3.31 × 3.31 × 3.31 *mm*^3^. The preprocessing is carried out using SPM12 and DPARSFA (Chao-Gan and Yu-Feng, 2010). The first 10 acquired rs-fMRI volumes of each subject were initially discarded before any further processing to ensure magnetization equilibrium. The remaining 130 volumes were then corrected for the staggered order of slice acquisition that was used during echoplanar scanning. The correction ensures the data on each slice correspond to the same point in time. To further reduce the effects of nuisance signals, regression of ventricle and WM signals as well as six head-motion profiles was performed. rs-fMRI images were then normalized to the MNI space with resolution of 3.31 × 3.31 × 3.31 *mm*^3^ (Wee et al., 2016). Participants with too much head motion are excluded. The normalized brain images are warped into automatic anatomical labeling (AAL) (Tzourio-Mazoyer et al., 2002) atlas to obtain 116 ROIs as nodes. By following common practice (Park, 2011; Leonardi and Van De Ville, 2013; Al-sharoa et al., 2018), the ROI mean time series are extracted by averaging the time series from all voxels within each ROI and then bandpass filtered to obtain multiple sub-bands as in Al-sharoa et al. (2018). After the preprocessing steps, we obtained the normal samples $X^{\left(1\right)}\in {\mathbb{R}}^{130\times 116\times 38}$ and eMCI samples $X^{\left(2\right)}\in {\mathbb{R}}^{130\times 116\times 44}$.

### 5.2. Classification

Almost every subject in ADNI dataset has several (≈6) individual rs-fMRI data series, that is, a patient might be scanned several times during a period of time. Usually, a random rs-fMRI data are selected and enters the processing step (Zhang et al., 2015). This random selection may cause several problems. Since the number of train data is very low, a small alteration in the samples could drastically change the set of input parameters in order to achieve the highest accuracy. Also achieving high-quality results with a classifier does not guarantee its effectiveness on other datasets even with fine-tuning the parameters, since the training set may contain outliers and unidentified corrupted data. In order to show that the proposed framework is less sensitive against the choice of different permutations of data, we have selected 18 different random permutations (i.e., each permutation contains a different rs-fMRI series, for each subject) and tested two state of the art eMCI classification methods on them: *HON* (Chen et al., 2016) and *k*−*SICE* (Zhang et al., 2015). We have used five evaluation measures: accuracy (ACC), sensitivity (SEN), Youden's index (YI), F-score, and balanced accuracy (BAC). The detailed definitions of these five statistical measures are provided in equation (Table 1), where TP, TN, FP, and FN denote the true positive, true negative, false positive, and false negative, respectively, and precision = $\frac{TP}{TP+FP}$ and recall = $\frac{TP}{TP+FN}$. In this article, we treat the eMCI samples as positive class and the NC samples as negative class.

**Table 1:** Definitions of five statistical measurement indices.

**Measurement**	**Definition**
Acc	$\frac{TP+TN}{TP+FP+TN+FN}$
SEN	$\frac{TP}{TP+FN}$
YI	*SEN*+*SPE*−1
F-Score	$2\times \frac{precession\times recall}{precesion+recall}$
BAC	$\frac{1}{2}\left(SEN+SPE\right)$

#### 5.2.1. Classification Performance

After fine-tuning the input parameter set for each method, the classification accuracy measure (ACC) shows that for 16 out of 18 different *random* selected datasets, our approach performs better than k-SICE the same also holds for 15 datasets comparing to HON, that is, in 88.8% of datasets the proposed method works better than k-SICE, and in 83.3% of datasets, it works better than FON. The highest classification accuracy (86.59%) is achieved with the proposed method in the 15th sample data. The highest accuracy for the HON (84.15%) is achieved in the 14th, and the highest accuracy for the SICE method (85.37%) is achieved in the 6th sample data. As it was mentioned before, being stable when the input dataset changes is a very important aspect for a classifier, in order to measure the stability, the standard deviation of accuracy along with other measures is calculated. The standard of accuracy for the proposed method is 0.64 times less than HON and 1.73 times less than k-SICE method. Similar results hold for other classification measures as well.

**Figure 3:**
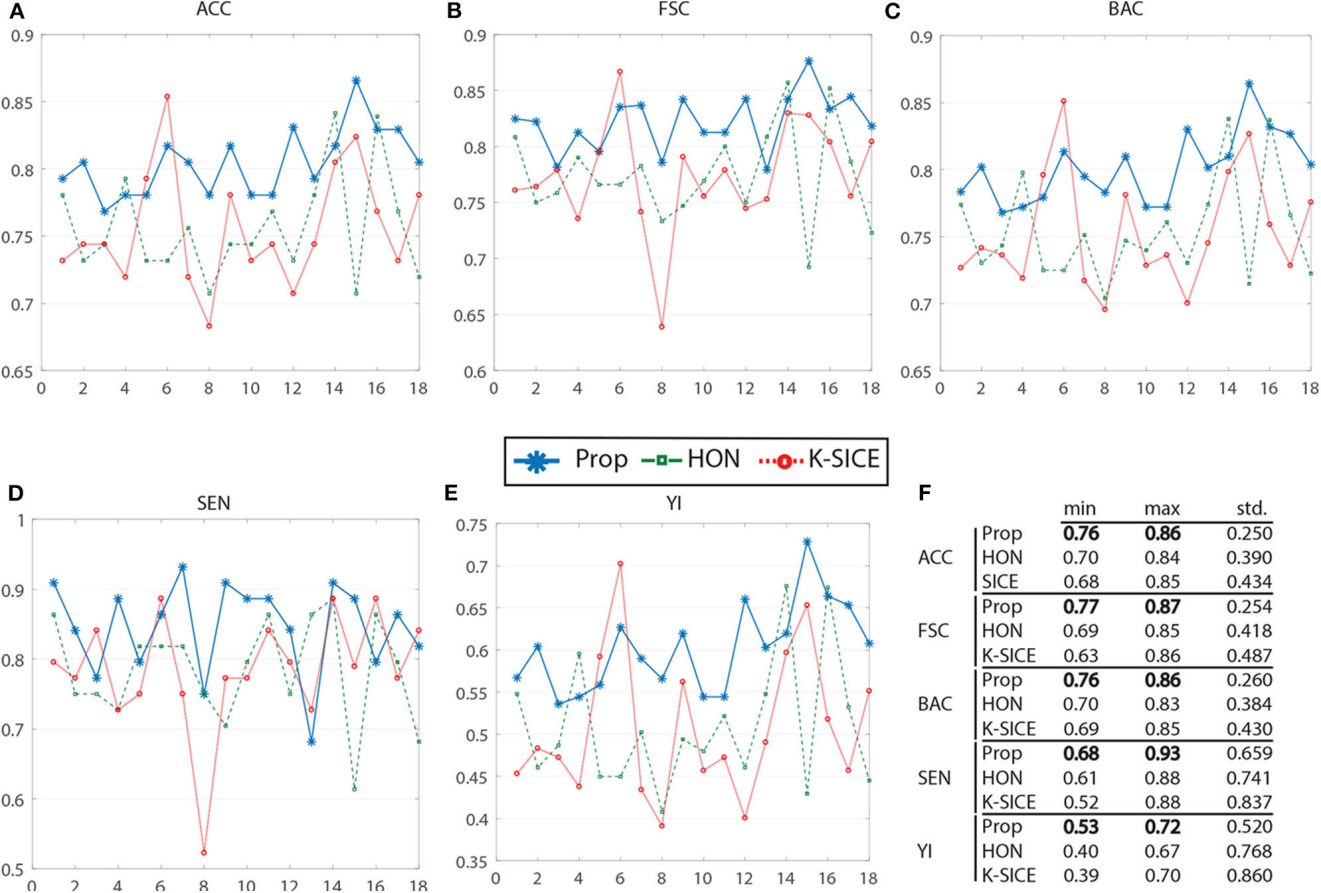
Comparison of proposed method (Prop) with K-SICE and HON applied on 18 different dataset permutations in five different classification evaluation measures. **(A–E)** show *accuracy, F-Score, balanced accuracy, sensitivity*, and *Youden Index*, respectively, along with the maximum, minimum, and standard deviation of each one presented in the embedded table **(F)**.

Figure 3 shows the performance of these three methods in all five measurements. For a better demonstration, Table 2 provides the average of several classification measurements scores for all dataset permutations. As it can be seen in this table, the average accuracy of proposed method, which is 80.43%, is 4.77% higher than the next method HON and 4.86% better than k-SICE. It is noteworthy that the other two methods, that is, HON and SICE, show similar results in average. The average F-score of the proposed method is also higher than other two, which shows a balanced prediction for both classes. Having a higher sensitivity (SEN) score, which measures the proportion of actual positives that are correctly identified as such, shows that the proposed method works better in detecting eMCI subjects. The YI is a measure for evaluating the biomarker effectiveness and having a higher YI yields a more informative decision (Youden, 1950). Our YI score is roughly 1.2 times better that other two methods. Similar to F-score, having a higher Balanced Accuracy Score (BAC) yields more balanced predictions. It is also noteworthy that the proposed method have much less standard deviation in all five measurements, which indicates its effectiveness and robustness toward different datasets.

**Table 2:** The average of different classification measurements in all dataset permutations in percent.

**Method**	**ACC**	**F-Score**	**SEN**	**YI**	**BAC**
k-SICE	75.57	77.36	78.50	50.69	75.34
HON	75.66	77.44	78.40	50.89	75.44
Proposed	**80.43**	**82.20**	**84.60**	**60.20**	**80.09**

*Higher values are indicated by bold numbers*.

One other key aspect of the proposed classifier is that it works significantly faster that the other two, especially in the training process. Our method is more than 600 times faster than HON and 20 times faster than SICE. Having a huge execution time especially affects the parameter selection scheme since all these methods use cross-validation procedure in order to find the optimal parameters, which itself requires several runs of the algorithm.

### 5.3. Functional Connectivity Network

**Figure 4:**
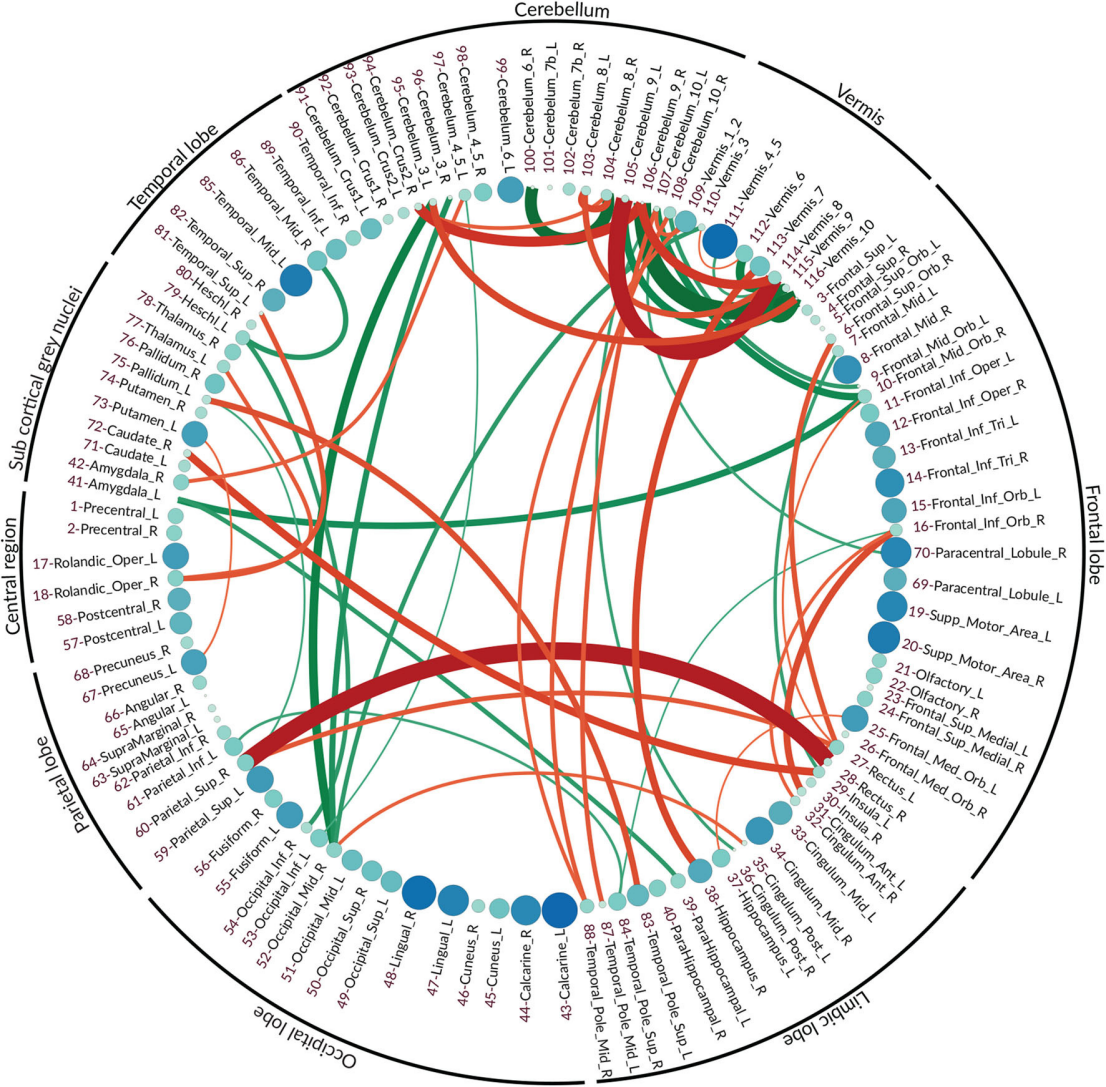
The difference graph. This graph is obtained via subtracting the functional connectivity of eMCI subjects from normal subjects. Each circle represents an ROI in AAL atlas, and the color and size of each circle are proportional to the graph clustering coefficient of the difference graph. Red: more activity in EMCI, green: less activity in EMCI.

The vector features for both normal and eMCI classes were obtained via the proposed method as it is described in section 4.3. Due to the aforementioned qualities of partial correlation, SICE is deployed in order to obtain the final FC. In order to better highlight the differences between normal and eMCI subjects, a difference graph *D* is constructed by subtracting the normal FC from the eMCI FC. This graph could be seen in Figure 4. The nodes of *D* show the ROIs according to the AAL atlas. The size of each node is proportional to its graph clustering coefficient, that is, the bigger node demonstrates higher activity in eMCI subjects in the corresponding ROI. Similar to nodes, the size of each edge is also proportional to the correlation between two ROIs. In addition, the edges are also color coded in a way that the green edges show the positive edges in *D* and the red edges show the negative edges in *D*. In this manner, the green edges demonstrate a decrease in activity between the corresponding nodes in eMCI subjects, and the red edges show increasing activity between corresponding ROIs in the eMCI subjects.

As it can be seen in the difference graph, the big nodes, that is, ROIs with higher activities, do not necessarily establish strong connections with the other nodes. As an obvious example, higher activities in lingual gyrus (ROI index: 47, 48) (He et al., 2007), calcarine sulcus (ROI index: 43, 44) (Bakkour et al., 2013; Brewer and Barton, 2014), supplementary motor area (ROI index: 19, 20) (Brewer and Barton, 2014; Jacobsen et al., 2015), and temporal_mid_L (ROI index: 85) (Kosicek and Hecimovic, 2013) are easily detectable. The majority of ROIs located in frontal lobe also show rather high activities compared to normal subjects (Dennis and Thompson, 2014; Salvatore et al., 2015).

Similar to the nodes, the strong edge between two ROIs does not necessarily require the nodes to be highly active in eMCI, although a strong edge does indicate high activities and FC between the two corresponding ROIs. The difference in graph shows a significant increase in connectivity between Rectus (ROI index: 28, 27 in frontal lobe) and Parietal_Sup_R (ROI index: 60 in parietal lobe) (Brickman et al., 2015; De Reuck et al., 2015), Frontal_Inf_Orb_R (ROI index: 16 in frontal lobe) and Cingulum_Ant (ROI index: 31, 32 in limbic lobe) (Perani et al., 2017), Insula_L, Temporal_Pole_Sup_L (ROI index: 29, 83 in limbic lobe) and Pallidum_R, Caudate_R (ROI index: 29, 83 in sub-cortical gray nuclei) (Watson et al., 2016). It can also be seen that within activities, frontal lobe also increased in patients with eMCI (Cai et al., 2015). There is a decrease in connectivity between Amygdala_L (ROI index: 41 in sub-cortical gray nuclei) with Frontal_Mid_Orb_R (ROI index: 10 in sub-frontal lobe) and ParaHippocampal_L (ROI index: 39 in sub-limbic lobe) (Ortner et al., 2016). The connectivity between Heschl_L (ROI index: 79 in temporal lobe) and two ROIs Temporal_Mid_R (ROI index: 86 also in temporal lobe) and Occipital_Inf_R (ROI index: 54 in occipital lobe) also decreased in eMCI (Steketee et al., 2016).

#### 5.3.1. Regarding the Cerebellum and Vermis

In fMRI data analysis and especially in AD studies, ROIs within the cerebellum and vermis are usually excluded since their role was regarded as insignificant (Sanz-Arigita et al., 2010; Zhang et al., 2011). Recent studies have shown that the traditional assumption that cerebral area is essential only to the coordination of voluntary motor activity and motor learning is not valid and indicates the significant role of the cerebellum in nervous system function, cognition, and emotion (Jacobs et al., 2017).

As it can be seen in the difference graph that we obtained, ROIs within cerebellum and vermis are highly active and both their Intra and interconnections are noticeable. There is increased FC between the limbic lobe, especially Hippocampus_R, Temporal_Pole_Mid (ROI index: 38, 87, 88) and cerebral areas in eMCI patients. Also, the connectivity between occipital lobe, especially occipital_mid_R (ROI index: 52), the frontal lobe, especially in frontal_mid_orb (ROI index: 9,10), and cerebral areas seems to decrease in patients with eMCI.

## 6. Conclusion

In this article, we proposed a tensor framework for eMCI diagnosis and FC construction. There are two main issues associated with rs-fMRI analysis and in particular eMCI diagnosis. The first is that the majority of state-of-the-art fMRI classification techniques use the FC matrix as the feature for their discriminant function; hence, they have to deal with many challenges that are associated with FC calculations. The second comes from the fact that FC networks are among the best tools for studying brain activities, but the stationary and dynamic FC conflict and the fact that the majority of methods belonging to these paradigms work only with one sample would lead to vastly different brain networks. Therefore, we developed a tensor framework, which is able to directly use the samples in classification without the need for any FC calculations and is also able to calculate a general FC network that considers the time-varying nature of rs-fMRI signals since it works in the data-dependent HOSVD-domain and is able to consider all subjects within a class in order to obtain these connectivities. The proposed method is not only fast, but it also outperforms state-of-the-art techniques.

One possible drawback of this framework is the need for HOSVD calculation for both classes in each test phase. Although this problem is negligible in eMCI classification (since the number of samples is not high), it could be time consuming for larger datasets. In order to resolve this issue, incremental HOSVD calculations may be deployed that will accelerate the calculations.

## Data Availability Statement

All datasets presented in this study can be downloaded through ADNI website (http://adni.loni.usc.edu). Additional information about the subjects used in this study can be found in the Supplementary Material.

## Author Contributions

AN and MR contributed to the design and implementation of the research, to the analysis of the results, and to the writing of the manuscript. Both authors contributed to the article and approved the submitted version.

## Conflict of Interest

The authors declare that the research was conducted in the absence of any commercial or financial relationships that could be construed as a potential conflict of interest.

## Footnotes

^1^http://adni.loni.usc.edu
